# Facile Synthesis of Microsphere-like Co_0.85_Se Structures on Nickel Foam for a Highly Efficient Hydrogen Evolution Reaction

**DOI:** 10.3390/mi14101905

**Published:** 2023-10-05

**Authors:** John Anthuvan Rajesh, Jae-Young Kim, Soon-Hyung Kang, Kwang-Soon Ahn

**Affiliations:** 1 School of Chemical Engineering, Yeungnam University, Gyeongsan 712-749, Republic of Korea; 21500915@ynu.ac.kr (J.A.R.); kjh9805140@naver.com (J.-Y.K.); 2 Department of Chemistry Education, Chonnam National University, Gwangju 500-757, Republic of Korea; skang@jnu.ac.kr

**Keywords:** Co_0.85_Se, hydrothermal, selenization, hydrogen evolution reaction, electrocatalytic activity

## Abstract

Microsphere-shaped cobalt selenide (Co_0.85_Se) structures were efficiently synthesized via a two-step hydrothermal process. Initially, cobalt hydroxide fluoride (Co(OH)F) microcrystals were prepared using a hydrothermal method. Subsequently, Co_0.85_Se microsphere-like structures were obtained through selenization. Compared to Co(OH)F, the microsphere-like Co_0.85_Se structure exhibited outstanding catalytic activity for the hydrogen evolution reaction (HER) in a 1.0 M KOH solution. Electrocatalytic experiments demonstrated an exceptional HER performance by the Co_0.85_Se microspheres, characterized by a low overpotential of 148 mV and a Tafel slope of 55.7 mV dec^−1^. Furthermore, the Co_0.85_Se electrocatalyst displayed remarkable long-term stability, maintaining its activity for over 24 h. This remarkable performance is attributed to the excellent electrical conductivity of selenides and the highly electroactive sites present in the Co_0.85_Se structure compared to Co(OH)F, emphasizing its promise for advanced electrocatalytic applications.

## 1. Introduction

The development of efficient and sustainable energy conversion technologies is of paramount importance in addressing the growing global energy demand and mitigating environmental concerns [1,2]. Among the various renewable energy sources, hydrogen gas (H_2_) holds immense promise as a clean and high-energy-density fuel [3,4]. Its production through water electrolysis, particularly via the hydrogen evolution reaction (HER), has garnered significant attention due to its potential to provide a clean and versatile energy carrier [5,6].

Traditionally, the alkaline HER has been described using the Volmer–Heyrovsky mechanism or the Volmer–Tafel mechanism [4,5,6]. However, both of these mechanisms are limited by sluggish kinetic processes, which hinder their overall efficiency. To address this challenge, researchers have turned to electrocatalysts as a means of enhancing HER performance. Over the past decade, electrocatalysts based on transition metals, including hydroxides, sulfides, selenides, phosphides, nitrides, and carbides, have gained widespread recognition in pioneering research efforts [7,8,9,10,11,12]. Among these, transition metal selenides have emerged as particularly promising candidates [13,14,15,16,17]. Notably, cobalt-based selenides have gained significant interest due to their exceptional catalytic properties and remarkable electrical conductivity [17,18,19,20,21].

In recent decades, morphology engineering has emerged as a pivotal strategy for enhancing the electrocatalytic properties of cobalt-based selenide materials. For instance, Lan et al. conducted pioneering work in shape-controlled synthesis, allowing the fabrication of various CoSe_2_ morphologies, including wires, spheres, and rods, by modulating the amount of NH_4_F. Among these diverse morphologies, CoSe_2_ microspheres demonstrated exceptional promise, achieving an impressively low overpotential of 167 mV at 10 mA cm^−2^ and a Tafel slope of 38 mV dec^−1^ for the HER in acidic media [22]. Similarly, Shen et al. developed microsphere-like Co_0.85_Se structures, incorporating sulfur into their design, resulting in a remarkable electrocatalytic performance for the HER [23]. Additionally, Hao et al. introduced a novel HER electrocatalyst composed of 1D ultrafine cobalt selenide nanowires intertwined with 2D Ti_3_C_2_T_x_ MXene nanosheets, achieving a low overpotential of only 84 mV, an impressive Tafel slope of 56 mV dec^−1^, and exceptional cycling stability [24]. Despite these remarkable achievements, it remains essential to manipulate the morphology and structure of cobalt-based selenide materials in order to fully unlock their potential and further enhance their catalytic performance.

In this context, we present a facile and effective method for the synthesis of microsphere-like cobalt selenide (Co_0.85_Se) structures on nickel foam (Ni-foam), designed to enhance their catalytic activity in the HER. Utilizing a two-step hydrothermal approach, we successfully engineered microsphere-like Co_0.85_Se structures. Electrocatalytic investigations demonstrated the remarkable HER performance of these microsphere-like Co_0.85_Se structures, characterized by an impressively low overpotential of 148 mV at a current density of −10 mA cm^−2^ and a Tafel slope of 55.7 mV dec^−1^. The outstanding performance of Co_0.85_Se structures can be attributed to their exceptional electrical conductivity, along with the abundance of electroactive sites, which surpass those found in Co(OH)F. This combination of features positions Co_0.85_Se structures as a promising electrocatalyst for efficient and sustainable hydrogen production.

## 2. Experimental Section

### 2.1. Chemicals and Reagents

Cobalt nitrate (Co(NO_3_)_2_·6H_2_O), ammonium fluoride (NH_4_F), sodium selenite (Na_2_SeO_3_), hydrazine hydrate (N_2_H_4_), and potassium hydroxide (KOH) were acquired from Sigma Aldrich Chemicals, Seoul, Republic of Korea. Ethyl alcohol (C_2_H_6_O) was procured from DUKSAN Pure Chemicals Co. Ltd., Seoul, Republic of Korea. Deionized water (DI H_2_O) was utilized for the washing process. All chemicals and reagents were employed without additional purification.

### 2.2. Synthesis of Co(OH)F Microcrystals on Ni-Foam

In the typical one-step preparation method, Co(NO_3_)_2_·6H_2_O (50 mM, 0.297 g) and NH_4_F (150 mM, 0.1111 g) were dissolved in 20 mL of DI H_2_O by stirring for 10 min. The resulting clear, pink-colored solution was then transferred into a 100 mL Teflon-lined, stainless-steel autoclave. Following that, a thoroughly cleaned Ni-foam substrate with a size of 10 mm × 50 mm was immersed into the reaction solution, and a hydrothermal treatment was conducted at 120 °C for 12 h using an electric furnace. Once the reaction had finished, the autoclave was allowed to cool down naturally to room temperature. The resultant pink-colored Co(OH)F microcrystals coated on the Ni-foam were collected and washed multiple times using DI H_2_O and C_2_H_6_O. Subsequently, the synthesized sample was left to dry overnight in an electric oven at 70 °C.

### 2.3. Conversion of Co(OH)F to Co_0.85_Se Microcrystals

In the second step, the Co(OH)F microcrystal precursor underwent a transformation into its corresponding Co_0.85_Se morphology through a selenization process. To provide further details, 0.2 g of Na_2_SeO_3_ and 1 mL of hydrazine hydrate were added to a solution containing 20 mL of DI H_2_O, along with the previously prepared Co(OH)F microcrystal precursor deposited on the Ni-foam substrate. This mixture was subsequently transferred into a 100 mL Teflon-lined, stainless-steel autoclave and subjected to heating at 120 °C for 12 h. Following the completion of the selenization process, the autoclave was allowed to cool to room temperature. The resulting black-colored sample was thoroughly rinsed with DI H_2_O and C_2_H_6_O and subsequently dried in an electric furnace for 12 h at 70 °C.

### 2.4. Materials Characterization

X-ray diffraction (XRD) analysis was employed to determine the crystalline phase, utilizing a PANalytical X’Pert Pro instrument with Cu Kα radiation (λ = 1.54060 Å), operating at a voltage of 40 kV and a current of 30 mA. X-ray photoelectron spectroscopy (XPS) scans were conducted using a Thermo Scientific K-Alpha ESCA spectrometer, employing monochromatized Al Kα radiation. The morphology was examined through field emission scanning electron microscopy (FESEM) using a Hitachi S4800 instrument, as well as field emission transmission electron microscopy (FETEM) using an FEI Tecnai G2 F20. The elemental composition of the samples was analyzed using energy-dispersive X-ray spectroscopy (EDX) with Oxford Instruments, Abington, UK.

### 2.5. Electrochemical Measurements

The electrochemical HER was evaluated using a standard three-electrode setup on the IVIUMSTAT electrochemical workstation. The counter electrode was a graphite rod, mercury/mercury oxide (Hg/HgO) was employed as the reference electrode, and the working electrode consisted of the Co_0.85_Se microcrystals. For comparison, the precursor Co(OH)F microcrystal electrocatalyst was also tested for HER in 1 M KOH. Linear sweep voltammetry (LSV) was conducted at a scan rate of 5 mV s^−1^ from −1.0 to −1.8 V vs. Hg/HgO. The Tafel slope values were obtained from their respective polarization curves. The durability of the as-prepared Co(OH)F and Co_0.85_Se electrocatalysts was evaluated using the chronopotentiometry (CP) technique at a constant current density of −10 mA cm^−2^ over a duration of 24 h. The electrochemical double-layer capacitance (Cdl) of the electrocatalysts was determined from the cyclic voltammetry (CV) curves obtained at various scan rates within the potential range of −0.3 to −0.35 V vs. Hg/HgO. Electrochemical impedance spectroscopy (EIS) was carried out over a frequency range of 100 kHz–0.01 Hz with an AC voltage of 5 mV. In this work, all electrocatalytic studies were conducted at room temperature.

In this study, the Hg/HgO electrode was converted into a reversible hydrogen electrode (RHE) according to the Nernst equation [25]:

*E* (RHE) = *E* (Hg/HgO) + 0.0591 × pH + *E^o^* (Hg/HgO) (1)

where *E* (RHE) represents the converted potential, *E* (Hg/HgO) denotes the potential experimentally measured against the Hg/HgO reference electrode, and *E^o^* (Hg/HgO) stands for the standard potential of Hg/HgO at 25 °C (0.098 V).

## 3. Results and Discussion

**Figure 1:**
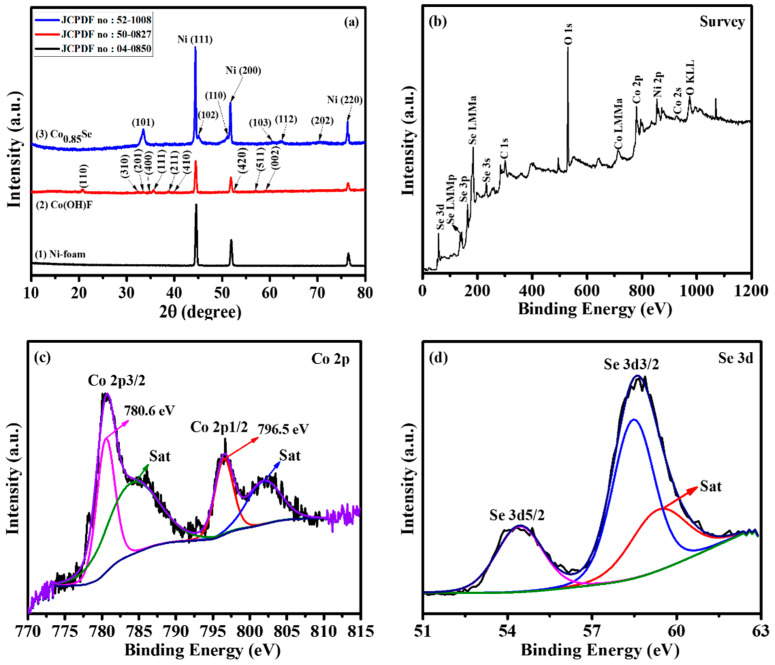
(**a**) XRD pattern of Ni-foam substrate, Co(OH)F microcrystals, and Co_0.85_Se microspheres. XPS characterization of Co_0.85_Se microspheres: (**b**) survey spectrum, (**c**) Co 2p, and (**d**) Se 3d.

After synthesis, the crystalline phases of the as-prepared electrocatalysts were analyzed using XRD analysis. The strong XRD peaks at 44.6, 51.9, and 76.4◦ 2θ were assigned to the Ni-foam substrate (Figure 1(a1)). As depicted in Figure 1(a2), the diffraction peaks of the first-step hydrothermal product closely match those of Co(OH)F (JCPDF no: 50-0827). The diffraction peaks at approximately 20.8°, 32.3°, 33.5°, 34.8°, 35.6°, 38.8°, 39.9°, 52.7°, 56.9°, 59.1°, and 61.5° correspond to the (110), (310), (201), (400), (111), (211), (410), (420), (511), (002), and (601) planes of orthorhombic Co(OH)F, confirming their good agreement with previous literature reports [26,27,28]. During the second hydrothermal step, the Co(OH)F structure underwent conversion into Co_0.85_Se through a low-temperature selenization process (Figure 1(a3)). The observed peaks at 33.5°, 45.0°, 51.1°, 60.5°, 62.5°, and 70.2° can be attributed to the (101), (102), (110), (103), (112), and (202) planes of hexagonal Co_0.85_Se [20,21,23]. Notably, no additional diffraction peaks were detected, affirming the high purity of the Co_0.85_Se phase. These XRD findings conclusively demonstrate the successful synthesis of the Co_0.85_Se electrocatalyst through the selenization of Co(OH)F.

To investigate the chemical states of the microsphere-like Co_0.85_Se, we performed further XPS measurements (Figure 1b–d). The survey spectrum of Co_0.85_Se (Figure 1b) clearly indicates the presence of peaks corresponding to C 1s, O 1s, Co 2p, and Se 3d. The C 1s and O 1s peaks originate from the reference material and inevitable surface oxidation of the sample, respectively. The Co 2p spectrum in Figure 1c reveals two main peaks at 780.6 and 796.5 eV, corresponding to Co 2p3/2 and Co 2p1/2, respectively [29,30,31]. These values are consistent with the reported literature on Co_0.85_Se structures. Additionally, two satellite peaks at 784.5 eV and 802.2 eV are attributed to the Co 2p3/2 and Co 2p1/2 peaks (Figure 1d). The deconvolution of the Se 3d spectrum displays two contributions at 54.9 and 58.8 eV, corresponding to Se 3d5/2 and Se 3d3/2, respectively [29,30,31]. These contributions are related to the metal–selenide bond.

**Figure 2:**
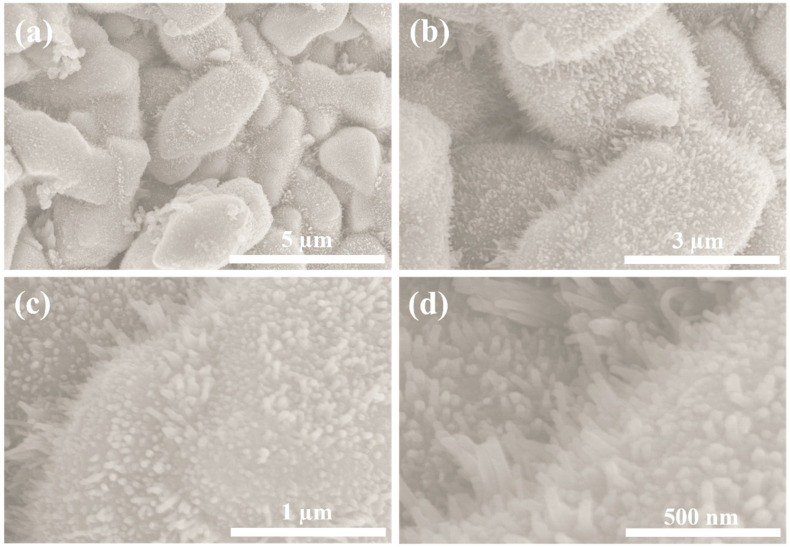
(**a**–**d**) FESEM images of microsphere-like Co_0.85_Se structures at different magnifications.

**Figure 3:**
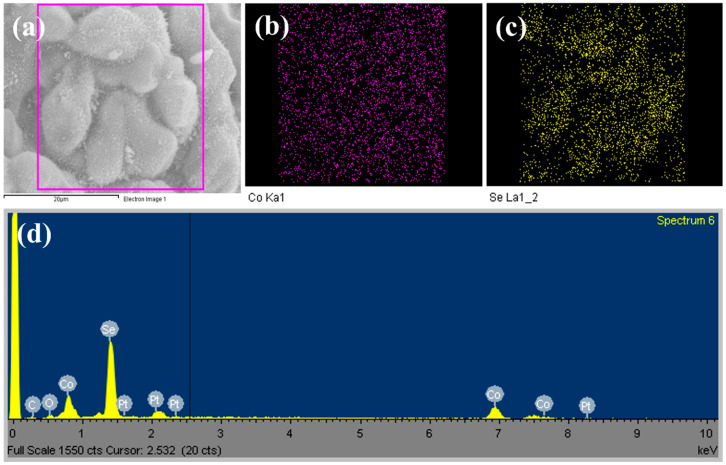
EDS mapping images of Co_0.85_Se microspheres: (**a**) electron image, (**b**) Co, (**c**) Se, and (**d**) EDX spectrum.

The FESEM technique was employed to analyze the morphology of the as-prepared Co(OH)F and Co_0.85_Se structures. Figure S1 displays the FESEM images of the Co(OH)F microcrystals synthesized using the hydrothermal method. The overall morphological view indicates that the prepared microcrystals exhibit a combination of tetrahedral, octahedral, and dodecahedral faces, each approximately 1 μm in size, consistent with our previous reports [26,32]. In Figure S2, the EDAX spectrum and mapping reveal the presence of cobalt, oxygen, and fluoride elements, without any impurities, thus confirming the high purity of the Co(OH)F microcrystals. Figure 2 illustrates the FESEM images of the selenized Co(OH)F product. As shown in Figure 2a–d, the morphology of Co_0.85_Se closely resembles that of Co(OH)F, indicating the preservation of the hierarchical microcrystal structure during the selenization process. It can be observed that the Co_0.85_Se structure displays spike-like features on its surface, which are characteristics resulting from the selenization process. The spherical appearance of the Co_0.85_Se structure is attributed to the presence of these spike-like structures. Furthermore, the elemental mappings and the EDX spectrum in Figure 3 reveal a homogeneous distribution of Co and Se elements within the microsphere-like Co_0.85_Se structure.

**Figure 4:**
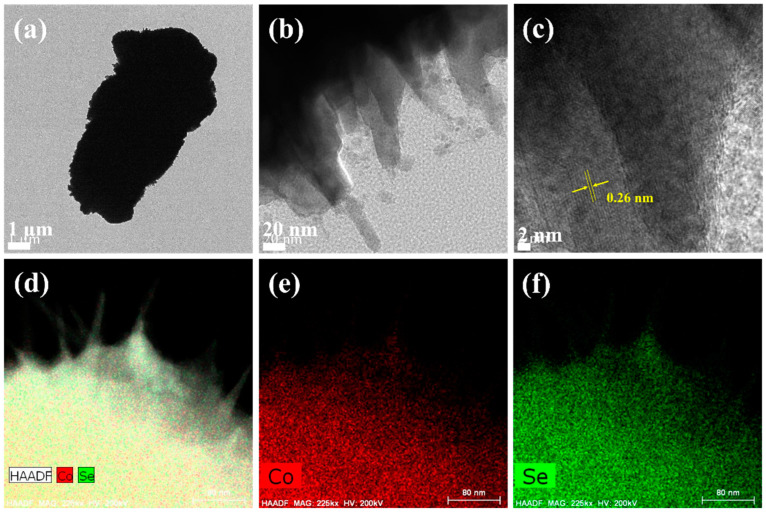
(**a**–**c**) TEM and HRTEM images of Co_0.85_Se microspheres. EDS mapping images of (**d**) HAADF, (**e**) Co, and (**f**) Se.

The detailed morphology and composition of the microsphere-like Co_0.85_Se structure were elucidated using HRTEM and EDX analyses. A representative TEM image of Co_0.85_Se, shown in Figure 4a, indicates its microsphere-like morphology. The high-magnification TEM image in Figure 4b reveals the presence of spikes on the surface, consistent with FESEM analysis (Figure 2b,c). The high-resolution TEM (HRTEM) image in Figure 4c further demonstrates the crystalline nature of the microsphere-like structure. The calculated interlayer distance was measured as 0.26 nm, corresponding to the (101) crystal plane of the Co_0.85_Se structure [20,21,33]. Elemental mapping images derived from the HAADF image (Figure 4d–f) demonstrate the uniform distribution of Co and Se within the sphere-like morphology. The EDX spectrum, shown in Supporting Figure S3, further confirms that the composition of the Co_0.85_Se structure consists of Co and Se. The overall physicochemical characterization demonstrates the successful preparation of microsphere-like Co_0.85_Se structures from Co(OH)F microcrystals through a selenization process.

**Figure 5:**
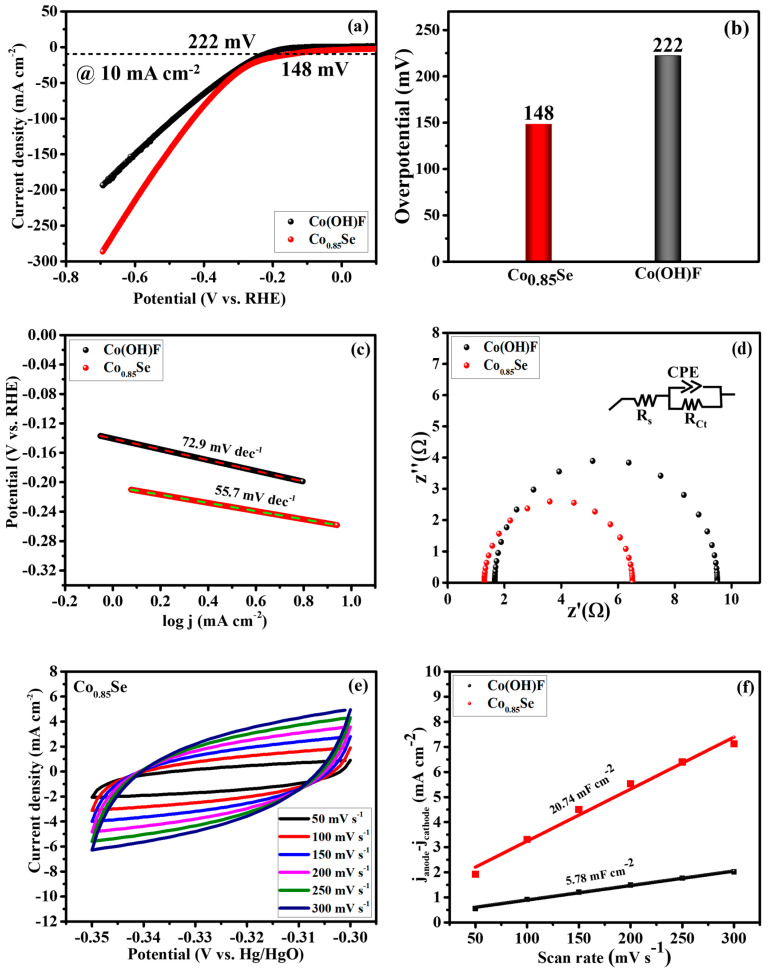
(**a**–**d**) LSV curves, overpotential, Tafel plot and Nyquist plots of Co(OH)F and Co_0.85_Se electrocatalysts. (**e**) Typical CV curves within a non-faradic region at different scan rates for Co_0.85_Se electrocatalyst. (**f**) The fitted Cdl plot of Co(OH)F and Co_0.85_Se electrocatalysts. All the measurements were conducted at room temperature.

The electrocatalytic properties of Co(OH)F and Co_0.85_Se catalysts were comprehensively investigated for the HER. Prior to linear sweep voltammetry (LSV) measurements, each electrode was activated through CV in a potential range of −0.1 to −0.8 V vs. Hg/HgO at a scan rate of 50 mV s^−1^ for 50 cycles. Subsequently, after CV activation, the polarization curves of the Co(OH)F and Co_0.85_Se electrocatalysts were measured at a scan rate of 5 mV s^−1^. Figure 5a,b illustrate the LSV curves and overpotential profiles of the Co(OH)F and Co_0.85_Se electrocatalysts in a 1.0 M KOH electrolytic solution. The LSV curves provide compelling evidence of Co_0.85_Se’s superior HER activity, requiring a lower overpotential of 148 mV compared to Co(OH)F (222 mV) to achieve a current density of 10 mA cm^−2^. To gain deeper insights into the catalytic activity of the prepared samples, Tafel slopes were derived from the LSV curves. Figure 5c shows the Tafel plots, where Co_0.85_Se exhibits a notably lower Tafel value of 55.7 mV dec^−1^ compared to Co(OH)F’s 72.9 mV dec^−1^. Importantly, the overpotential and Tafel value of the prepared Co_0.85_Se microspheres (148 mV, 55.7 mV dec^−1^) were found to be significantly lower than those of other CoSe-based materials, including p-CoSe_2_/CC (138 mV, 83 mV dec^−1^) [18], Co_7_Se_8_ (472 mV, 59.1 mV dec^−1^) [34], Co_0.85_Se@NC (230 mV, 125 mV dec^−1^) [35], CoSe_2_/CNTs (186 mV, 52 mV dec^−1^) [36], CoSe_2_/C-HRD (157 mV, 110 mV dec^−1^) [37], CoSe_2_/MoSe_2_ (218 mV, 76 mV dec^−1^) [38], Ni-doped CoSe_2_ (172 mV, 32.4 mV dec^−1^) [39], B-CoSe_2_/CC (153 mV, 85 mV dec^−1^) [40], CoSe_2_@MoSe_2_ (183 mV, 43.37 mV dec^−1^) [41], and MoSe_2_-CoSe_2_ (148 mV, 45 mV dec^−1^) [42]. The remarkable electrocatalytic behavior of Co_0.85_Se can be attributed to the strong metallic bonding between Co^2+^ and Se^2−^, which accelerates the dissociation of H_2_ from H_2_O. The electroactivity of Co_0.85_Se is primarily centered on the Se active sites, as this facilitates the weakening of the thermodynamic energy barrier for the HER [43]. In this reaction process, Co_0.85_Se, where Co^2+^ carries a positive charge and Se^2−^ carries a negative charge, can readily adsorb H_2_O molecules following the ‘Volmer–Heyrovsky’ pathway. Co^2+^ readily accepts electrons from Se^2−^, leading to reduction through the adsorption of H_2_O on the surface. Furthermore, the electronegative nature of Se^2−^ makes it conducive for the decomposition of H_2_O into H* and OH^−^ ions, followed by the dissociation of OH^−^ ions, resulting in the regeneration of H* and the continuation of H_2_ evolution [44]. It has been reported that the H* generated through the Volmer pathway forms a weak bond with Se, and the delocalization of positive and negative charges between Co and Se significantly enhances the adsorption and desorption of H*, thus promoting the HER [14,45,46].

To elucidate the superior HER performance of Co_0.85_Se, we conducted EIS measurements. The charge transfer resistance (Rct) behavior of each electrocatalyst was determined from the Nyquist plots, and the fitted graph with an equivalent circuit is presented in Figure 5d. The analysis of the graph reveals that Co_0.85_Se exhibits a significantly lower Rct value of 6.5 Ω compared to Co(OH)F (9.56 Ω), confirming faster HER kinetics in the KOH solution, which contributes to its superior activity.

Furthermore, to assess the excellent performance of Co_0.85_Se, we estimated Cdl values to determine the electrochemical active sites via CV measurements within a non-Faradic region, in the potential range of −0.3 to −0.35 V vs. Hg/HgO, with a scan rate of 50–300 mV s^−1^ (Figure 5e). For comparison, typical CV curves for Co(OH)F were provided in Figure S4, and their corresponding Cdl values were calculated by plotting the current density (J anodic–J cathodic) at the potential of −0.325 V vs. the scan rate (Figure 5f). As anticipated, the Cdl value of Co_0.85_Se (20.74 mF cm^−2^) was found to be three times higher than that of Co(OH)F (5.78 mF cm^−2^), indicating an increase in the electrochemical active sites, which inherently facilitates the performance of metal active sites in the electrolytic solution for HER.

**Figure 6:**
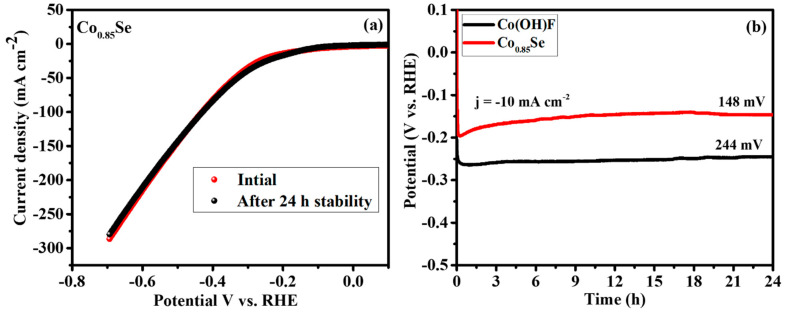
(**a**) LSV curves of Co_0.85_Se, obtained initially and after 24 h of the chronopotentiometry stability study, and (**b**) long-term stability comparison of Co_0.85_Se and Co(OH)F electrocatalysts at a constant potential of −10 mA cm^−2^ for 24 h. All the measurements were conducted at room temperature.

The stability of the electrocatalyst is a crucial factor ensuring a stable HER process. Consequently, the long-term stability of the electrocatalysts Co_0.85_Se and Co(OH)F was tested using chronopotentiometry at a constant potential for 24 h, delivering a current density of −10 mA cm^−2^. The corresponding LSV curves, obtained both initially and after 24 h of stability testing, are depicted in Figure 6a. The successive CV curves for the Co_0.85_Se electrode before and after 24 h confirm the durability of the catalyst. The chronopotentiometry graph shown in Figure 6b clearly illustrates the long-term stability of Co_0.85_Se and Co(OH)F at constant potentials of −0.148 V and −0.244 V, respectively, while delivering a current density of −10 mA cm^−2^. From this comprehensive investigation, it can be concluded that Co_0.85_Se’s remarkable HER activity stems from its excellent fast electron transfer transport ability, larger electrochemical active sites, and long-term stability.

## 4. Conclusions

In this study, we successfully synthesized microsphere-like Co_0.85_Se structures on Ni-foam using a facile two-step hydrothermal method. These Co_0.85_Se structures exhibited remarkable catalytic activity in the HER when compared to Co(OH)F, signifying their potential for advanced electrocatalytic applications. The as-prepared microsphere-like Co_0.85_Se structures exhibited a low overpotential of only 148 mV and a Tafel slope of 55.7 mV dec^−1^. Notably, the Co_0.85_Se electrocatalyst showcased remarkable long-term stability, maintaining its activity for over 24 h of continuous operation. The remarkable performance of the Co_0.85_Se structures can be attributed to their superior electrical conductivity, coupled with the presence of a high number of electroactive sites when compared to Co(OH)F. These findings underscore the considerable promise of Co_0.85_Se microsphere-like structures on Ni-foam as a highly efficient and stable catalyst for the HER.

## Data Availability

The data presented in this study are available on request.

## Supplementary Materials

The following supporting information can be downloaded at: https://www.mdpi.com/article/10.3390/mi14101905/s1. Figure S1. (a–d) FESEM images of Co(OH)F microcrystals at different magnifications. Figure S2. (a) FESEM electron image and (b) corresponding EDX spectrum of Co(OH)F microcrystals. EDX elemental maps of (c) cobalt, (d) oxygen, and (e) fluorine. Figure S3. (a) HRTEM HAADF image and (b) corresponding EDX spectrum of the Co_0.85_Se. Figure S4. Typical CV curves at different scan rates in the non-Faradaic region for Co(OH)F electrocatalyst.
