# Supplementary material for: Facile Synthesis of Microsphere-like Co0.85Se Structures on Nickel Foam for a Highly Efficient Hydrogen Evolution Reaction
Source: Micromachines (Basel). 2023 Oct 5;14(10):1905. doi: 10.3390/mi14101905 (PMC10608889; doi:10.3390/mi14101905)
Supplement: Supplementary file 1 [file micromachines-14-01905-s001.zip › micromachines-2638174-supplementary.pdf]

Facile Synthesis of Microsphere-Like $Co_{0.85}Se$ Structures on Nickel Foam for a Highly Efficient Hydrogen Evolution Reaction

John Anthuvan Rajesh,[1] Jae-Young Kim,[1] Soon-Hyung Kang,[2] and Kwang-Soon Ahn[1]*

[a]*School of Chemical Engineering, Yeungnam University, Gyeongsan 712-749, S. Korea*

[b]*Department of Chemistry Education, Chonnam National University, Gwangju 500-757, South Korea*

*Corresponding author: E-mail address: kstheory@ynu.ac.kr;

Tel: +82-53-810-2524; Fax: +82-53-810-4631

Figure S1

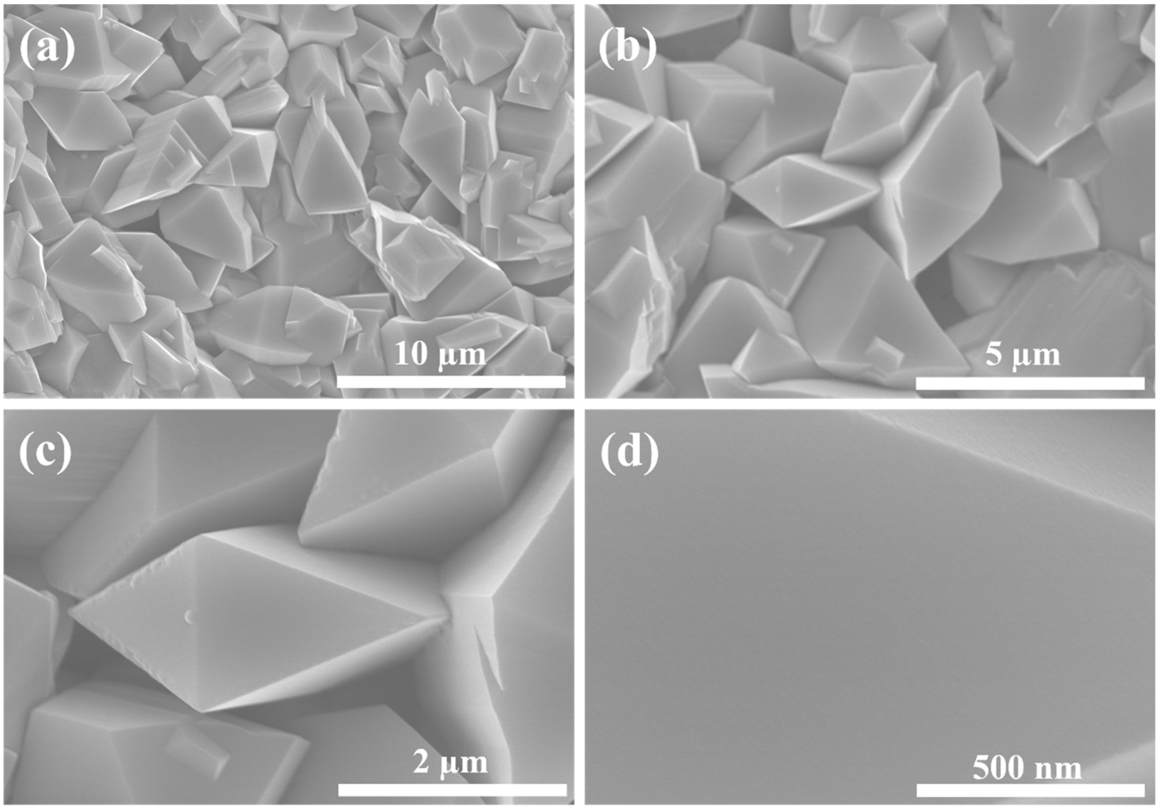

Figure S1. (a-d) FESEM images of Co(OH)F microcrystals at different magnifications.

Figure S2

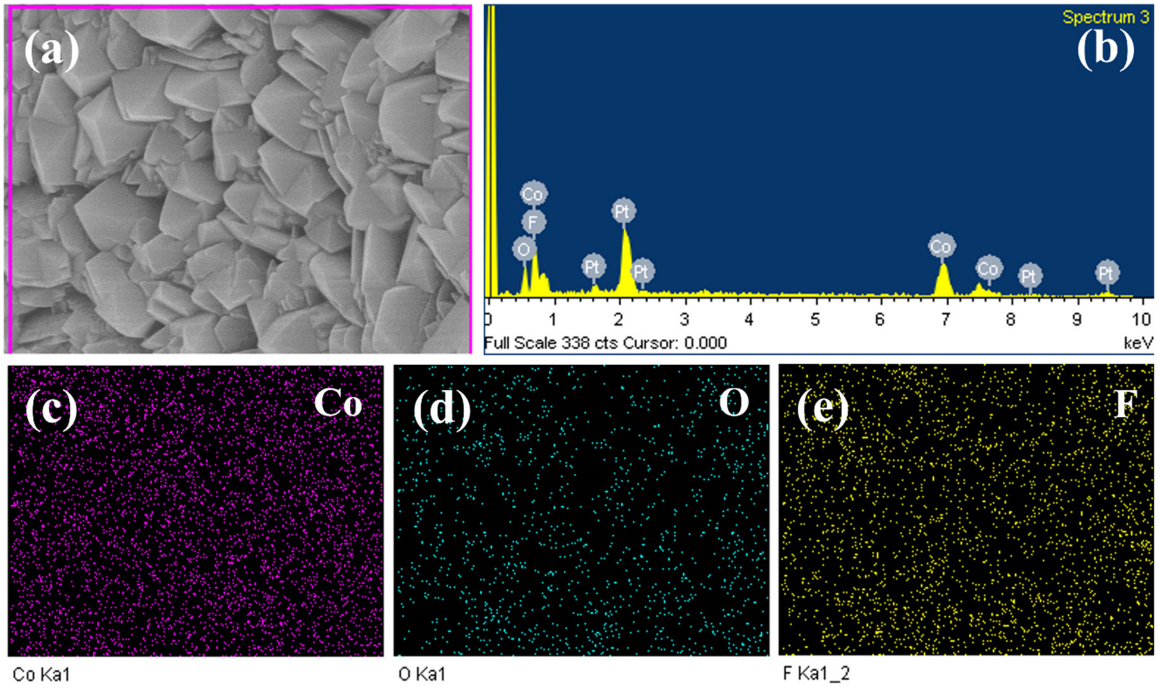

Figure S2. (a) FESEM electron image and (b) corresponding EDX spectrum of Co(OH)F microcrystals. EDX elemental maps of (c) cobalt, (d) oxygen, and (e) fluorine.

Figure S3

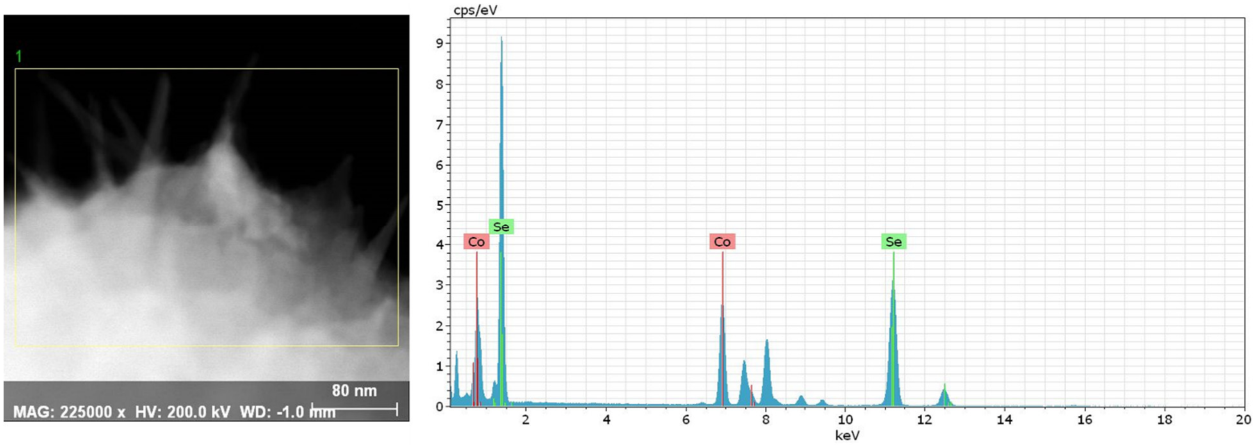

Figure S3. (a) HRTEM HAADF image and (b) corresponding EDX spectrum of the $Co_{0.85}Se$.

Figure S4

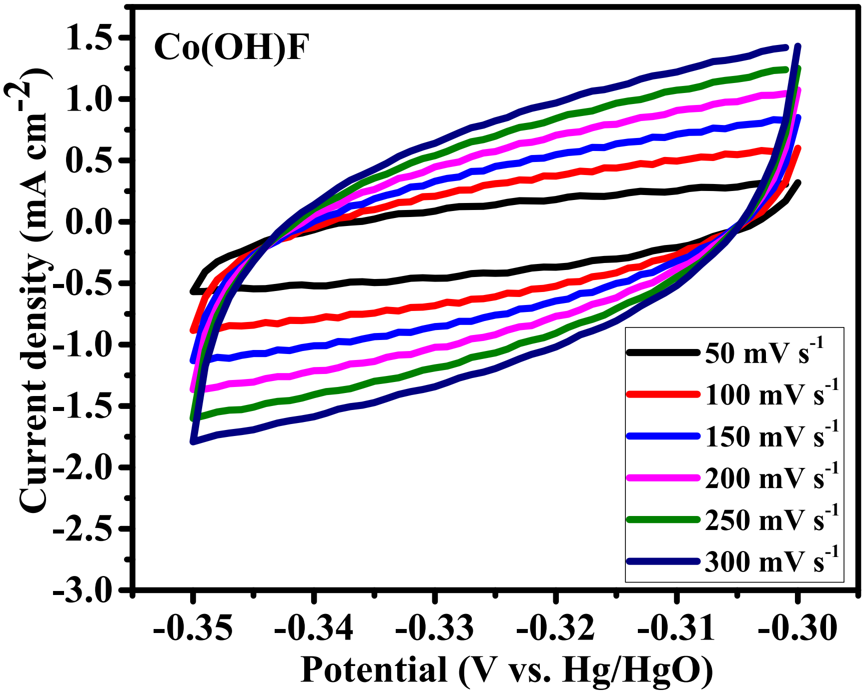

Figure S4. Typical CV curves at different scan rates in the non-Faradaic region for Co(OH)F electrocatalyst.
